# Opportunity to view the starry night sky is linked to human emotion and behavioral interest in astronomy

**DOI:** 10.1038/s41598-024-69920-4

**Published:** 2024-08-20

**Authors:** Rodolfo Cortes Barragan, Andrew N. Meltzoff

**Affiliations:** 1 Institute for Learning & Brain Sciences, University of Washington, Seattle, WA 98195 USA; 2 Department of Psychology, University of Washington, Seattle, WA 98195 USA

**Keywords:** Psychology and behaviour, Human behaviour

## Abstract

Prior to the modern era, the stars in the night sky were readily visible across the globe, but light pollution has created disparities in the opportunity to see these astronomical objects with the naked eye. This alteration may measurably impact human behavior. We hypothesize that light pollution is related to the development of people’s interest in astronomy, which often serves as a “gateway” to science more broadly. In a state-by-state analysis, we used location information to examine astronomy interest data for millions of US residents. Results show that, among populations with low light pollution, a feeling of “wonder about the universe” is prevalent (*r* = 0.50). We found that this human emotion mediates the association between low light pollution and behavioral interest in astronomy. Although the effects of light pollution on astronomy, biology, ecology, and health are well-known, the present work demonstrates that light pollution is also relevant to human scientific behavior, with broad implications for science education and society.

## Introduction

In addition to being a problem for ground-based astronomy^1–3^, anthropogenic light at night is transforming world ecologies^4–6^. Even in locations hundreds of kilometers away from the main sources of artificial light—the urban areas—the propagation of light through the atmosphere degrades the visibility of the night skies^7^. This artificial light has consequences for biological processes as diverse as animal migration^8^, pollination^9^, circadian rhythms^10^, and gene expression^11^. Artificial light at night also affects human health^12,13^. There is a growing consensus that this light can be considered a conventional environmental pollutant^14^. A major underexplored issue is how light pollution, which blocks humans’ natural views of the starry night sky, might affect human behavior and even scientific curiosity.

Prior to incandescent street and building lighting, starlight served as a fixture of the nighttime for human groups around the globe^15^. Human civilizations relied on the stars to inform agricultural, navigational, and cultural practices^16^. In the industrial era, stars in the night sky began to disappear from the daily experience of urban populations^17,18^. This anthropogenic phenomenon is growing rapidly, and the stars are becoming a rare sight for many human populations^2,7,19^. These changes in the visibility of the starry night sky may plausibly affect human psychology and behavior, especially in relation to interest in one of humanity’s oldest sciences—astronomy.

We hypothesized that there would be a specific psychological mediator between the loss of the visible starry night sky and dampened behavioral interest in astronomy: A feeling of “wonder” about the universe. Indeed, historic and modern astronomers have informally reported that their own inspiration for studying the universe was seeing the starry night sky and feeling “wonder” about the universe^20–24^. These narratives are consistent with theories in psychological science, which propose that wonder—a human emotion combining curiosity, amazement, and elation—is a strong motivator of human behaviour^25^. Wonder involves recognizing gaps in current understanding and endeavoring to bridge those gaps by acquiring new knowledge^26^. Here we hypothesize that the loss of the visible starry night sky reduces the opportunities for humans to feel wonder about the universe and dampens interest in astronomy, a “gateway science”^27^.

## Results

**Figure 1:**
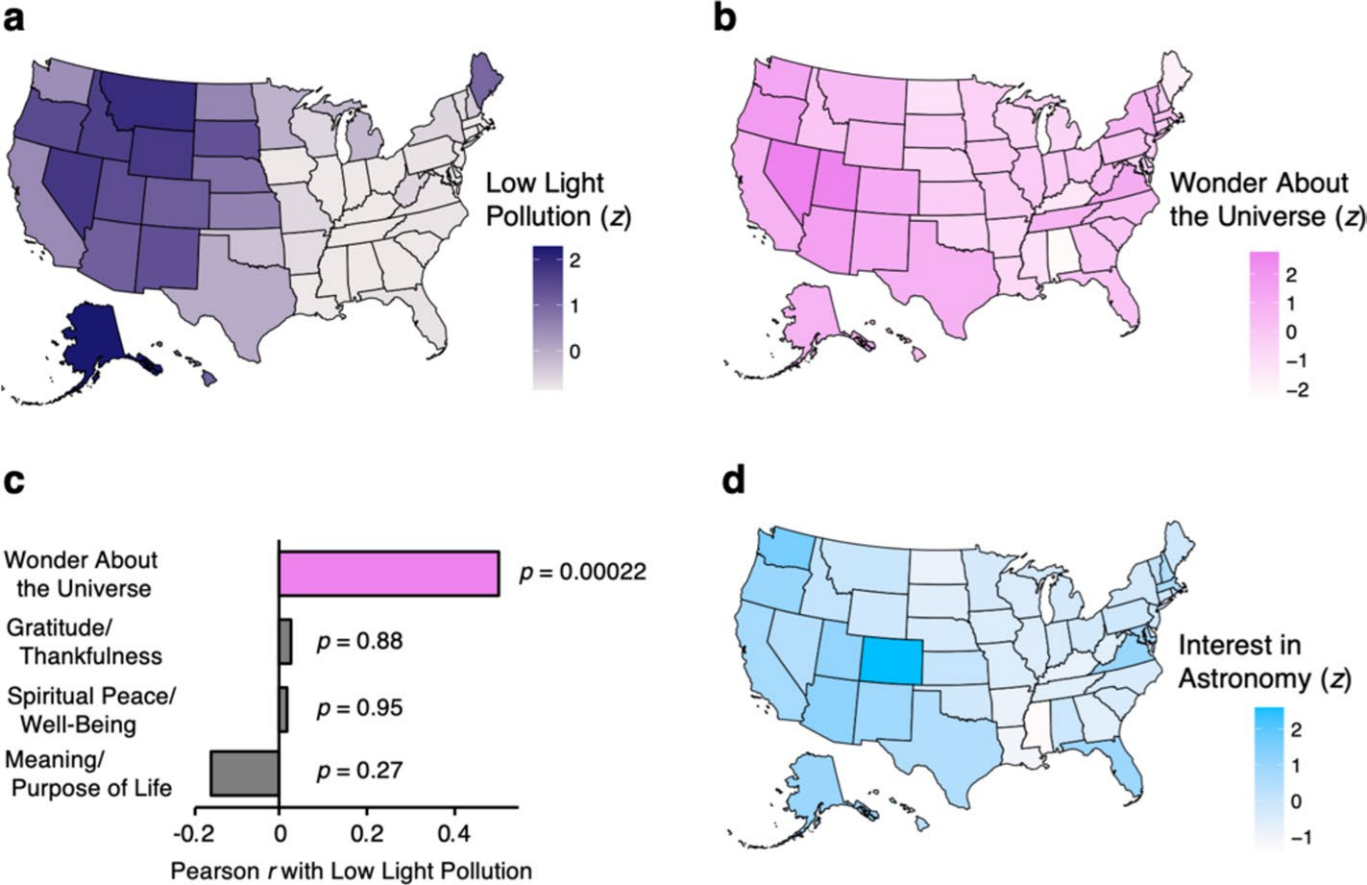
State maps of low light pollution, wonder about the universe, astronomy interest, and correlations between measures. (**a**) Map of US states showing low light pollution^28^, purple (darker purple indicates less light pollution). (**b**) Map of US states showing respondents’ sense of wonder about the universe from the Pew Research Center^29^, pink (darker pink indicates more wonder about the universe). (**c**) Pearson correlations between low light pollution and wonder about the universe, and between low light pollution and other psychological emotions. (**d**) Map of US states showing composite measure of behavioral interest in astronomy, blue (darker blue indicates more interest in astronomy). All measures are *z*-standardized and defined in the Supplemental Information (also see Methods).

At the level of the individual person, there are no known measures of exposure to light pollution. Thus, we examined physical measurements of artificial night sky brightness, as reported at the state level in the US by Falchi and colleagues^28^. Consistent with their call to use their dataset to explore issues in the behavioral sciences^2,7,28^, we focused on gradients of light pollution that allow the naked human eye to view nighttime stars at the zenith, i.e., low light pollution (Fig. 1a; also see Supplementary Information, Sect. 1.1 for measurement details).

Contemporaneously with Falchi et al.^28^ collecting measurements, the Pew Research Center collected data from 35,071 US respondents using a rigorous survey that captured psychological measures from a representative sample of residents in each state^29^. This Pew survey included the psychological question “How often do you feel a deep sense of wonder about the universe?” measured on a 5-point Likert scale (Supplementary Information, Sect. 1.2). This measure varied by state across the US (Fig. 1b).

### Light pollution and wonder about the universe

Our analyses of these two independent datasets show a positive association at the state level between low light pollution and feelings of wonder about the universe, *r* = 0.50, *p* < 0.001 (Fig. 1c). Importantly, the Pew survey also measured three other psychological questions that served as controls in the present study, because they were collected in randomized order on the same individuals who answered the question about wonder (specifically, Pew collected measures of gratitude/thankfulness, spiritual peace/well-being, and meaning/purpose of life, Supplementary Information, Sect. 1.2). These psychological variables also varied by state across the US (Supplementary Fig. 1), but there was no significant association (*p*s > 0.27) between low light pollution and any of these control covariates (Fig. 1c).

For completeness, we note that for a sample of 50 (*df* = 48), a Pearson *r* coefficient would need to meet or exceed *r* = 0.28 [absolute value] to achieve *p* < 0.05. Also, because the low light pollution data were skewed, necessitating interpretive caution, we re-calculated the *r* using a dichotomous median split for the light pollution data, and found that the correlation remained significant, see Supplementary Information, Sect. 2.

### Wonder about the universe and astronomy interest

Psychological perspectives hold that emotions such as wonder can drive learning and exploration^30,31^. We hypothesized that wonder about the universe would be associated with behavioral interest in astronomy, and developed eight measures of such interest at the state level (Supplementary Information, Sects. 1.3–1.9).

The first measure was seeking to learn about astronomy. Using the billions of searches conducted on Google^32^, we found that states with more wonder about the universe also have a higher proportion of total internet searches for “astronomy,” *r* = 0.52, *p* < 0.001 (Supplementary Information, Sect. 1.3 and Supplementary Fig. 2a).

Second, we examined people’s interest in contributing to astronomical knowledge. We found a positive association between state-level wonder about the universe and the percent of the state population that submits data to the NSF-NOIRLab’s citizen scientist project Globe at Night^33^, *r* = 0.40, *p* = 0.004 (Supplementary Information, Sect. 1.4 and Supplementary Fig. 2b).

Third, interest in astronomy may be seen more broadly in popular culture, and Nobel laureate astrophysicist Kip Thorne consulted on a scientifically-grounded film depiction of exoplanetary exploration, *Interstellar*^34^. State-level wonder was positively associated with the state-level proportion of peoples’ web video searches for *Interstellar*, *r* = 0.50, *p* < 0.001 (Supplementary Information, Sect. 1.5 and Supplementary Fig. 2c).

The fourth and fifth analyses were conducted on a measure of peoples’ interest in voyaging to another planet. Specifically, signing up to have their name inscribed on a chip and sent to Mars affixed to NASA’s *InSight* lander and *Perseverance* rover. State-level wonder about the universe was positively associated with the percent of the state population that submitted their name to voyage aboard *InSight*, *r* = 0.56, *p* < 0.001, and *Perseverance*, *r* = 0.65, *p* < 0.001 (Supplementary Information, Sect. 1.6 and Supplementary Fig. 2d,e).

Sixth, people can take concrete steps to send their physical selves into space by training to become an astronaut. In 2020, NASA invited astronaut applications from those with documented STEM backgrounds, and more than 12,000 applied to join this “Artemis Generation.” We submitted a Freedom of Information Act (FOIA) request to NASA, and they released to us the number of astronaut applications from each of the states. State-level wonder about the universe was positively associated with the percent of people from each state with STEM-related backgrounds who applied to become a NASA astronaut, *r* = 0.34, *p* = 0.017 (Supplementary Information, Sect. 1.7 and Supplementary Fig. 2f.).

Seventh, the James Webb Space Telescope (JWST) is NASA’s latest large strategic science mission^27^, and millions of people “follow” the telescope via Twitter (now X). We found that state-level wonder about the universe was positively associated with the percent of the state population that follows @NASAWebb, *r* = 0.53, *p* < 0.001 (Supplementary Information, Sect. 1.8 and Supplementary Fig. 2g).

Eighth, wonder about the universe may correlate with general interest in NASA, which can be indexed by subscriptions to the NASA Newsletter. Via a FOIA request, we received more than 975K de-identified records from NASA and found that state-level wonder about the universe was positively associated with the percent of the state population subscribed to NASA’s Newsletter, *r* = 0.37, *p* = 0.009 (Supplementary Information, Sect. 1.9 and Supplementary Fig. 2h).

**Figure 2:**
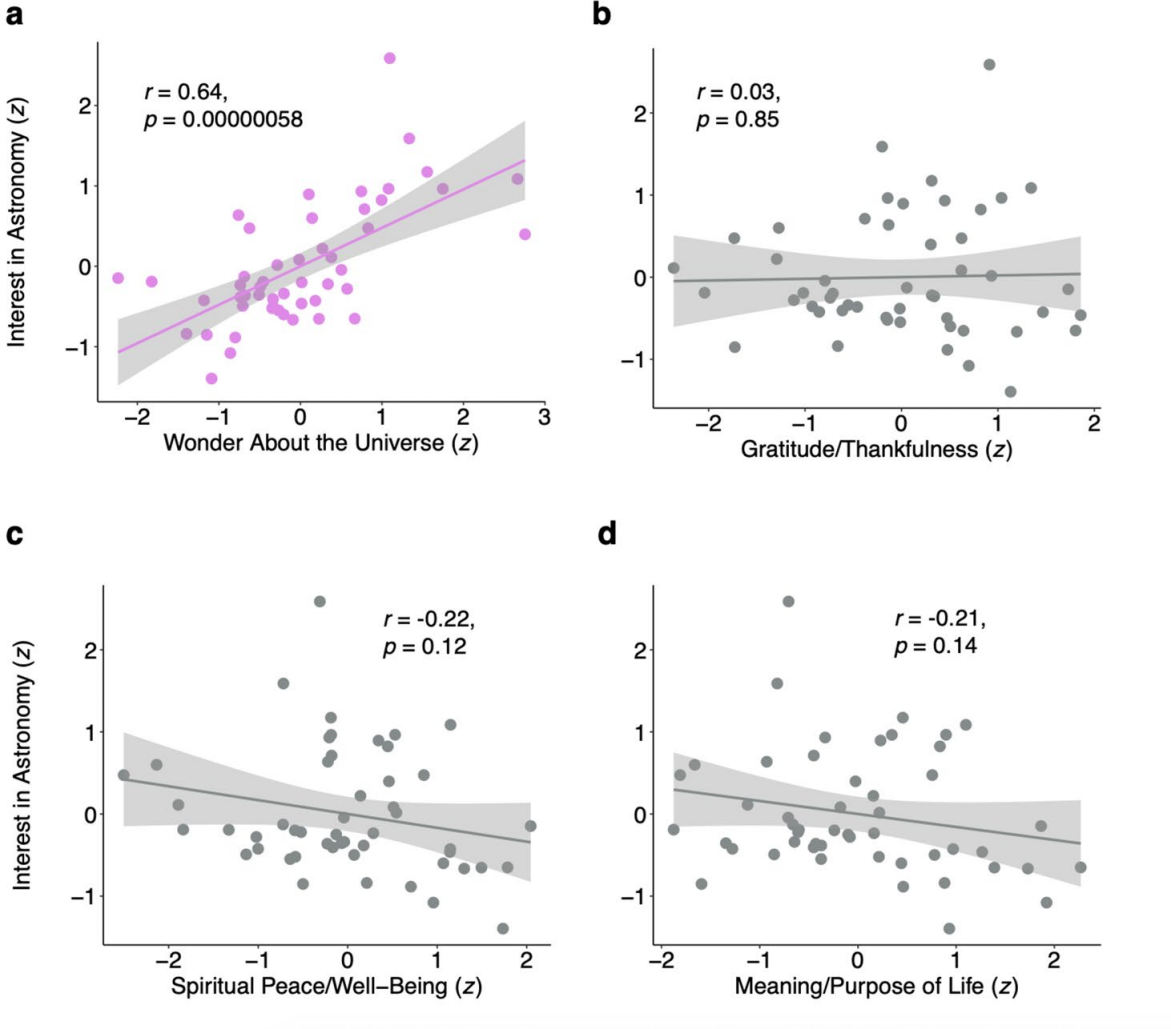
Scatterplots of association of wonder about the universe (and other emotions) with behavioral interest in astronomy (*N* = 50 states). (**a**) scatterplot showing significant correlation between wonder about the universe and composite measure of behavioral interest in astronomy (pink). (**b**–**d**) Scatterplots of the nonsignificant correlations between other control psychological measures and composite measure of behavioral interest in astronomy (gray). Shaded areas show 95% confidence intervals. All measures are *z*-standardized (see Methods).

Finally, we formed a composite measure of “behavioral interest in astronomy” (Fig. 1d; also see Supplementary Information, Sect. 1.10). As expected, there was a significant positive association between this state-level composite measure of behavioral interest in astronomy and state-level feelings of wonder about the universe (from the Pew survey), *r* = 0.64, *p* < 0.001 (Fig. 2a). Also as expected, there were no significant associations between behavioral interest in astronomy and the other variables from the Pew survey that served as controls in this study (Fig. 2b–d).

### Mediation analyses

We used statistical mediation models to assess the connections among light pollution, wonder about the universe, and behavioral interest in astronomy, using 95% confidence intervals with 5,000 bootstrap samples^35^. If a variable is demonstrated to serve as a “mediator,” it can be said to serve as a link between two other variables^35^ (see Methods). We examined whether wonder about the universe mediates between light pollution and behavioral interest in astronomy, even when controlling for other possible demographic covariates.

**Figure 3:**
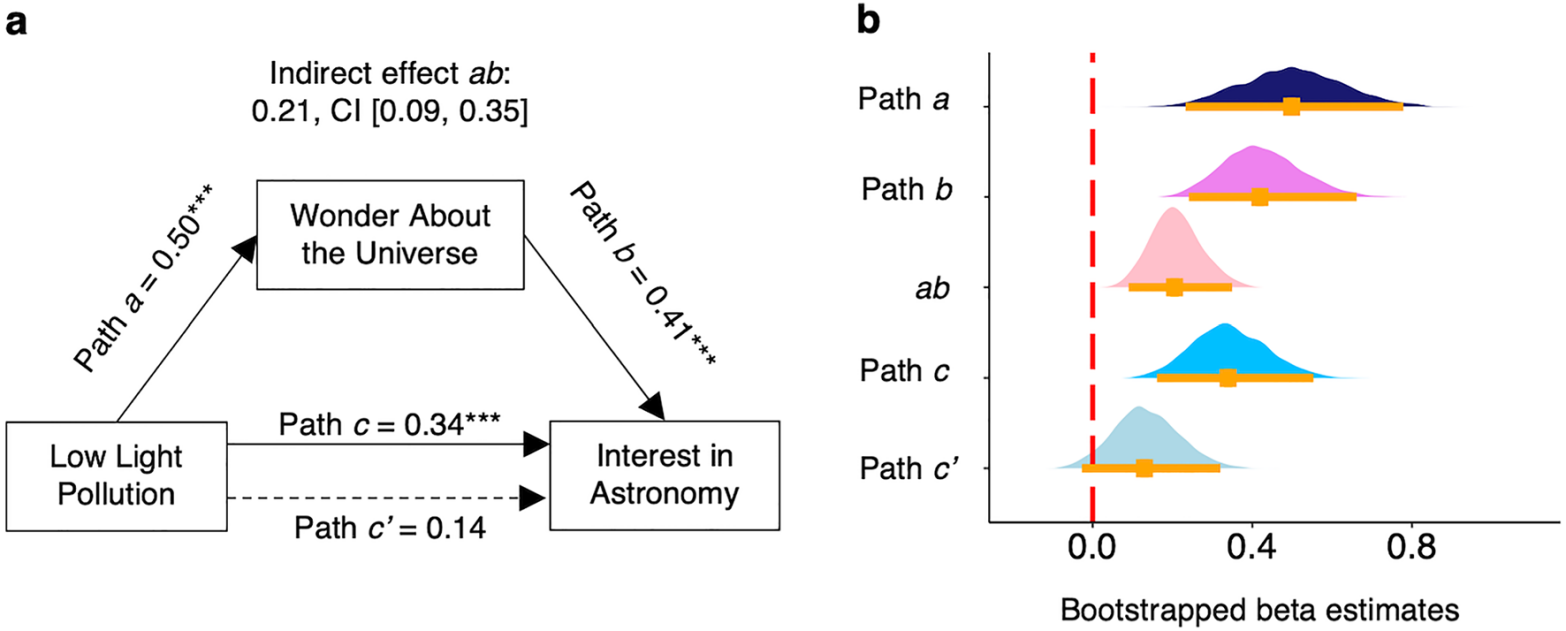
Mediation analysis. (**a**) Beta weights of the regression coefficients in mediation model showing low light pollution correlated with wonder about the universe (Path *a*) and wonder about the universe correlated with interest in astronomy (Path *b*), with the mediator (indirect) effect *ab* = 0.21. ***p < 0.001. (**b**) Smoothed density plots showing distribution of bootstrapped beta values produced in (**a**) by Hayes’ PROCESS Model 4^35^ with the following indicators: dashed vertical red line (beta value of zero), solid horizontal orange lines (95% confidence intervals of bootstrapped beta values), orange squares (medians of bootstrapped beta values).

In this mediation analysis, we tested whether the observed positive association between low light pollution and behavioral interest in astronomy, *r* = 0.45, *p* < 0.001, was mediated by wonder about the universe. As hypothesized, this effect was obtained: mediator effect *ab* = 0.21, 95% C.I. [0.09, 0.35] (Fig. 3a,b). Additionally, we examined potential confounders: education, poverty, race, population size, and population density (Supplementary Information, Sect. 1.11). When including these covariates, wonder about the universe was still a significant mediator between light pollution and behavioral interest in astronomy, mediator effect *ab* = 0.14, 95% C.I. [0.04, 0.28], and education was the only covariate associated with behavioral interest in astronomy (see Table 1). When we performed sensitivity analyses by analyzing the data using alternative models, we obtained substantively similar results (Supplementary Information, Sect. 3).

**Table 1:** Model summary, including covariates, predicting behavioral interest in astronomy (using Hayes’ PROCESS Model 4 procedure [35]).

Variable	*b*	*b SE*	*t*	*p*	95% C.I
Low light pollution	0.20	0.10	1.96	0.057	− 0.01–0.40
Wonder about the universe	0.27	0.09	3.09	0.0036	0.09–0.45
Covariates					
Education	0.43	0.12	3.57	0.00091	0.19–0.67
Poverty	0.07	0.12	0.57	0.57	− 0.17–0.30
Race	0.07	0.09	0.76	0.45	− 0.11–0.24
Population size	0.05	0.09	0.57	0.57	− 0.12–0.22
Population density	− 0.09	0.10	− 0.90	0.38	− 0.29–0.11

All measures were *z*-standardized. *N* = 50 states.

## Discussion

Light pollution has adverse consequences for biological and ecological systems^4–13,36^ as well as for the science of astronomy itself^1,2,37^. However, its effects on human behavior have been understudied. In this investigation, by combining data from the physical and psychological sciences, we showed that low light pollution is linked with the tendency of the population to feel wonder about the universe and to become interested in exploring it. This association exhibited some specificity—other psychological measures that had been measured in the same individuals at the same time in tandem with wonder about the universe were not associated with light pollution. The inclusion of multiple demographic covariates did not change the significant findings in the mediation model. That is, the mediation modelling still showed that a psychological factor (wonder about the universe) mediated the association between the physical environment (light pollution) and human behavior (interest in astronomy).

These findings suggest that humans’ loss of the opportunity to see the starry night sky is related to peoples’ behavior. From a human developmental and educational perspective, one is immediately drawn to the question of whether light pollution may influence the development of children’s and adolescents’ interest in science^38–41^. For example, seeing the starry night sky might prompt youths to think more about the physical universe, and to join relevant social/academic affinity groups to advance this knowledge as they develop academically^42,43^. One empirical study in central coastal California (a region of somewhat low light pollution) found that children as young as three- and four-years of age express curiosity about astronomical objects, asking caregivers for explanations (e.g., “Why do the stars shine?”)^44^. The opportunity to see the stars in the night sky could engender interest in astronomy and greater chances of gaining practice in everyday scientific thinking. Indeed, many astronomers report that, in their youth, seeing the night sky triggered a feeling of “wonder” about the universe, and that this was a motivator for them aspiring to become a scientist^20–24,45^. Further psychological work is needed to understand how children’s feelings of wonder and awe^46–48^ interact with early caregiver support^44,49^ and experiences in formal education^50,51^ to promote interest and engagement with astronomy, the natural world, and the process of science to address questions about the universe.

Additionally, data from diverse cultures are needed. This is especially the case for Africa, Latin America, Oceania, as well as European countries where light pollution is rapidly encroaching upon formerly pristine dark skies^52,53^. To combat this, light pollution researchers^54^ and architects^55^ are proposing interventions on lighting practices. Keeping light pollution in check may nurture “citizen science”^1,2,33^ which, itself, engenders public enthusiasm and financial support for science^27^. When interdisciplinary teams of scientists partner with communities most affected by light pollution (and other environmental challenges^56,57^) both science and society can benefit^58–62^.

We acknowledge two limitations of the present work. First, our findings are at the level of the US states and do not apply to individual people, which logically constrains the inferences that can be drawn^63,64^. Research that examines light pollution, wonder about the universe, and astronomy interest within the same individual, rather than at the state level, is needed. A second limitation is that correlations do not specify causality, and it remains possible that those who have an interest in astronomy, or a sense of wonder about the universe, may choose to live in low light pollution states. To more directly address causality, longitudinal studies of people as they develop intellectual interests, coupled with random-assignment educational interventions, would be valuable.

## Conclusion

Light pollution is recognized as an international concern. UNESCO representatives, government officials, and scientists jointly authored the La Palma Declaration, classifying the unpolluted night sky as an “inalienable right of humankind”^65^. Astronomers^1^ and light pollution researchers^66^ are calling for concrete actions to curb light pollution. As suggested here, increasing access to the starry night sky may also help to promote more equitable opportunities to feel wonder about the universe, which can motivate interest and engagement in science^20,67^. The present findings thus begin to suggest how light pollution is not only impacting biological and ecological processes, but also human behavior, science education, and society.

## Methods

### Analytic strategy

All analyses used the 50 US states^68^. All measures were *z*-scored to allow construction of the composite score, and to facilitate comparisons among the measures and between the states. Positive/negative *z*-scores indicate the values are above/below the central tendency of the distribution for that measure. Prior to mediation analyses, regression assumptions were checked and met.

### Mediation analysis method

In mediation analysis^35^, the predictor variable *X* is examined for its effect on mediator variable *M* (Path a), and *M* is examined for its effect on outcome variable *Y* (Path *b*). The mediator (indirect) effect, *ab*, is the product of Path *a* and Path *b*. The effect of *X* on *Y* is Path *c*. Path *c*’ is the effect of *X* on *Y* while accounting for *M*. Mediation was tested with Hayes’ PROCESS modeling tool (Model 4)^35^.

### Supplementary Information

Supplementary Information.

Supplementary Tables.

## Data Availability

The datasets that support the findings of this study are available as raw data (Supplementary Table 1) and as the z-transformed analytic dataset (Supplementary Table 2).

## Code availability

No special statistical code was used beyond that standardly embedded in R, RStudio, R packages (ggplot2, ggridges, usmap), SPSS 28.0.0.0, and Hayes’ PROCESS 4.2. Geocodio software was used to convert latitude and longitude to geographical units (for Globe at Night). FileMaker (Version 19) was used to code text data into geographical units (for JWST and the NASA Newsletter).
