# Supplementary material for: Opportunity to view the starry night sky is linked to human emotion and behavioral interest in astronomy
Source: Sci Rep. 2024 Aug 20;14:19314. doi: 10.1038/s41598-024-69920-4 (PMC11336222; doi:10.1038/s41598-024-69920-4)
Supplement: Supplementary file 1 — Supplementary Information. [file 41598_2024_69920_MOESM1_ESM.pdf]

Supplementary Information

Opportunity to view the starry night sky is linked to human emotion and behavioral interest in astronomy

Rodolfo Cortes Barragan[1*], Andrew N. Meltzoff[1,2*]

[1]Institute for Learning & Brain Sciences, University of Washington

[2]Department of Psychology, University of Washington

*Correspondence to: barragan@uw.edu; meltzoff@uw.edu

1. Measures

We describe below how each state-level measure was defined and calculated.

1.1. Low light pollution. Falchi et al.[1] published a comprehensive atlas of gradients of light pollution (physical measurements of the brightness of artificial light in the night sky) for each US state and advocated for these data to be used in the behavioral sciences (p. 1)[2]. Their data were published in bins of different magnitudes[1,2]: (i) 0 to 1.7 μcd/m^2 ("pristine skies"), (ii) 1.7 to 14 μcd/m^2 ("relatively unpolluted at the zenith but degraded toward the horizon"), (iii) 14 to 87 μcd/m^2 ("polluted sky degraded to the zenith"), (iv) 87 to 688 μcd/m^2 ("natural appearance of the sky is lost"), (v) 688 to 3,000 μcd/m^2 ("Milky Way loss"), and (vi) > 3,000 μcd/m^2 ("night adaptation is no longer possible for human eyes."). Falchi et al. (p. 4)[2] described brightness bins iii-vi as "light-polluted skies" and advocated for policy to protect bin i and bin ii[1-3]. Because of this, we focused on these two bins, and refer to them as "low light pollution," after Falchi et al. (pps. 4 and 10)[2].

For each bin, Falchi et al.[1] provided the percent of the US state population living within the bin (hereafter "percent population") as well as the percent of the land area of each state falling within each bin (hereafter "percent land area"). We found that the percent population was highly correlated with the percent land area, for both bin i (r = 0.81, p < 0.001) and bin ii (r = 0.73, p <0.001). We therefore computed a mean of percent population and percent land area. This resulted in a variable termed "mean percent population and percent land area with pristine skies" (i.e., bin i [0–1.7 μcd/m^2]) and, separately, another variable termed "mean percent population and percent land area with skies relatively unpolluted at the zenith but degraded toward the horizon" (i.e., bin ii [1.7–14 μcd/m^2]). To yield the analytic measure of low light pollution (i.e., bin i and bin ii, following Falchi et al.[1-3]), we first summed these two variables (M = 13.65%, SD = 16.26%, *Range* = 0%–50.65%), and second, z-standardized the values to form the analytic measure of low light pollution at the state level.

1.2. Wonder and other positive emotion measures. To calculate the state-level measures, we used the Pew Research Center's nationally representative survey (N = 35,071), which was conducted between June 4th and September 30th, 2014 with a minimum of 300 respondents per state[4]. The year of this Pew research survey (2014) was the same year as Falchi et al.'s physical measurements of light pollution[1,2]. Pew estimated that the survey was representative of 97% of the non-institutional population of the US and released the dataset to the public as an SPSS file: *Dataset - Pew Research Center 2014 Religious Landscape Study National Telephone Survey - Version 1.1 - December 1 2016.sav*.

Within this dataset, the specific questions relevant to the current paper are variables *qi4a*, *qi4b*, *qi4c*, *qi4d*. The columns in the Pew datafile provide respondents' numerical answers to specific questions from the telephone interviewer: "Now, thinking about some different kinds of experiences, how often do you: 'feel a deep sense of spiritual peace and well-being,' (*qi4a*), 'feel a deep sense of wonder about the universe,' (*qi4b*), 'feel a strong sense of gratitude or thankfulness,' (*qi4c*), 'think about the meaning and purpose of life'

(*qi4d*).” The order of the four questions was randomized in the survey administration. The interviewer read off response categories to participants as: “Would you say at least once a week (coded as ‘1’), once or twice a month (‘2’), several times a year (‘3’), seldom (‘4’), or never (‘5’).”

For analyses, we reverse-coded the data such that higher scores indicated more positive emotions. To derive the state-level measures, we followed Pew[4] and applied their weight variable, which was recommended by Pew as follows: “Analysts interested in national-level and state-level data…should weight the data using the variable WEIGHT”[4]. These state-level measures were *z*-scored, and are displayed in Fig. 1b and Supplementary Fig. 1.

1.3. Astronomy. Google Trends is a tool that indexes the popularity of search terms among trillions of searches (*https://support.google.com/trends/answer/4365533?hl=en*). The tool allows researchers to compare, in a selected country, the relative popularity of search terms between different subregions of the country. This involves a standardized process: When a query is made for a term, Google Trends examines a sample of Google’s dataset and—based on the relative popularity of the query term to all other search terms—displays a normalized score ranging from 0 to 100 for each subregion in country (in the current case, we examined the 50 US states).

On the Google Trends interface, we queried “astronomy” (field of study)—constraining the query to searches conducted between January 1, 2015 and December 31, 2022 (i.e., following the 2014 assessment of the focal predictors low light pollution[1] and wonder about the universe[4]). This approach is attentive to the timing of assessments, consistent with Hayes’ suggested practices for mediation analysis (p. 129-132)[5]. We ran seven different queries on seven days within a two-week period. As expected, these seven values were internally consistent, Cronbach’s alpha = 0.99. We used the mean of these seven values as the measure of state-level web searches for astronomy, and *z*-standardized the measure for analysis. Data are displayed in Supplementary Fig. 2a.

1.4. Globe at Night. For this state-level measure, we used NSF-NOIRLab’s Globe at Night project’s publicly available data (2015-2021). We calculated the number of Globe at Night submissions per US state (based on respondents’ latitude and longitude). These numbers were divided by the population of the state (see Section 1.11). The analysis was based on the *z*-standardized values. Data are displayed in Supplementary Fig. 2b.

1.5. Interstellar. To obtain the state-level measure, we repeated the process from Section 1.3, but for the term “Interstellar” (film) as a YouTube search query, for the same period as in Section 1.3, with data drawn on seven different days and averaged (Cronbach’s alpha = 0.99). Analysis based on the *z*-standardized values. Data are displayed in Supplementary Fig. 2c.

1.6. Insight, Perseverance. At the state-level, NASA released the number of people from each state who submitted their name to be sent to Mars on a chip affixed to *InSight* and *Perseverance*. State number totals were divided by the population of the state (see Section 1.11). Analyses of both measures are based on the *z*-standardized values. Data are displayed in Supplementary Fig. 2d,e.

1.7. Artemis. In March 2020, NASA opened applications for the astronaut “Artemis Generation.” Through a Freedom of Information Act (FOIA) request, we received the number of applications per state. Because NASA’s call for applications focused on people with STEM degrees, we divided the number of applications in a state by the number of individuals in that state with bachelor’s degrees of science, engineering, and related fields, as reported by the American Community Survey in 2020. Analysis used the *z*-standardized values. Data are displayed in Supplementary Fig. 2f.

1.8. JWST. We predetermined to examine the state-level sum of Twitter (now X) followers of @NASAWebb when its follower count reached 1.5M, which occurred on July 7th, 2022. Location data is publicly listed on each Twitter profile, for users who choose to self-report their location. We used www.followersanalysis.com to download these data and ran a script to code the data into states. We divided the total number of @NASAWebb followers of each state by the state population (see Section 1.11). The analysis used the *z*-standardized values. Data are displayed in Supplementary Fig. 2g.

1.9. Newsletter. We coded this state-level measure from a FOIA request, which yielded the self-reported locations of internet users who subscribed to NASA's Newsletter in 2019-2022. We coded the data and tabulated the number of subscribers per state and divided this number by the state population (see Section 1.11). The analysis used *z*-standardized values. Data are displayed in Supplementary Fig. 2h.

1.10. Composite of behavioral interest in astronomy. The state-level composite of astronomy interest was the average of the standardized values (*z*) for the eight measures listed in the foregoing Sections 1.3–1.9. The justification for combining the eight measures into a composite is: (i) the eight measures are correlated with each other (Supplementary Fig. 3), (ii) the Cronbach's alpha among the eight measures is high (Cronbach's alpha = 0.89), and (iii) principal component analysis suggested a one-factor solution, all factor loadings > 0.60.

1.11. State-level measures of education, poverty, race, population size, population density. The measures of the following five control covariates were drawn from three Census sources: (i) education (percent of population with a bachelor's degree), (ii) poverty (percent of the population in poverty), (iii) race (percent of population grouped as non-White, which was defined as follows): American Indian/Alaska Native, Asian, Black, Hispanic, Native Hawaiian/Pacific Islander, Multiracial, and Other), (iv) state population, and (v) population density. The three Census sources for this information were: (i) the American Community Survey (2014, i.e., the same year as the night sky brightness and wonder measures) (ii) the Annual Social Economic Supplement to the Current Population Survey (2014), and (iii) the Master Address File/Topologically Integrated Geographic Encoding and Referencing database (for land area measurements, in 2010). All measures were *z*-standardized for analysis. Supplementary Fig. 4 displays the zero-order correlations among these demographic variables and the composite behavioral interest in astronomy measure.

2. Alternative analysis of low light pollution and wonder about the universe. Regarding the main text section labelled "Light pollution and wonder about the universe," we note that the light pollution data deviate from the normal distribution. For this reason, we also used a simple dichotomous measure by sorting the 50 states into those below the median of low light pollution ($n = 25$) and those above the median of low light pollution ($n = 25$). We checked for statistically significant correlations between this dichotomous variable and each of the four emotions reported by Pew[4]. The results were substantively the same as reported with the continuous low light pollution variable in the main text. The positive association between light pollution (median split) and wonder about the universe was still significant, $r = 0.39$, $p = 0.005$, and none of the three other emotions was significantly associated with the dichotomous light pollution measure, *p*s > 0.12.

3. Sensitivity analyses for mediation model. As alternatives to the mediation models presented in the main text, we also examined three other mediation models.

First, we used the simple median split of the light pollution dataset to re-run the mediation model. With this dichotomous treatment, wonder about the universe continued to mediate the relation between low light pollution and behavioral interest in astronomy, mediator effect $ab = 0.33$, 95% C.I. [0.11, 0.60].

Second, we examined the impact of extreme values in the dataset by dropping three states (Colorado, Nevada, Utah) that could be considered outliers (i.e., > 2.50 *SD*s). When these were removed, wonder about the universe continued to mediate between low light pollution and behavioral interest in astronomy, mediator effect *ab* = 0.16, 95% C.I. [0.04, 0.33].

Third, although our hypothesis and the narratives from astronomers[6-11], suggested that the relation between low light pollution and astronomy interest would be mediated by the psychological experience of "wonder about the universe," one can test other alternative mediation models. Notably, low light pollution could be tested as the mediator between wonder about the universe and behavioral interest in astronomy. We conducted this analysis, and the results revealed no significant evidence for this alternative mediation, mediator effect *ab* = 0.07, 95% C.I. [-0.02, 0.17]. This is compatible with the core idea presented in the main text—that wonder about the universe, a psychological factor, is a mediator between low light pollution, an environmental predictor, and behavioral interest in astronomy, an outcome.

4. Study background and context. Here we provide a statement of the background motivation of the current research and more details about the development of the actual study, which was exploratory in nature and not preregistered.

This research had its initial roots in author R.C.B.'s experiences with seeing the night sky where he initially lived (semi-rural Mexico) and being struck by the absence of visible stars after moving to a light polluted area (central Los Angeles). Congruent with discussions by astronomers[6,11], he wondered whether experiences seeing the starry night sky might influence people's thoughts. The design of the present research was influenced by co-author A.N.M.'s work on psychological factors that motivate and/or dissuade individuals from engagement in STEM fields[12-14] and how curiosity and interest promote scientific thinking[15-17]. Also motivating us were our previous studies examining how environmental and cultural factors influence people's behavior[18-21].

The concrete design and execution of the current research was enabled by Falchi et al.'s 2019 publication of light pollution measures at the US state-level[1], Pew's data on wonder about the universe[4], and NASA's plans to launch JWST in 2021. We understood that Falchi et al.'s data afforded us the opportunity to test the hypothesis outlined in the main text, namely that wonder about the universe (as measured by Pew) was a psychological mediator between low light pollution (as measured by Falchi et al.) and interest in astronomy (behavioral measures we could assemble through FOIA requests to NASA and other sources).

We launched the planned study by submitting public records requests to NASA in 2021 and 2022 (respectively: FOIA Tracking Number 21-JSC-F-00478 for the Artemis astronaut applications, and FOIA Tracking Number 22-HQ-F-00824 for the data on the NASA Newsletter subscriptions). The other measures of people's behavioral interest in astronomy, including information about the 1.5M Twitter (now X) "followers" of the JWST (@NASAWebb), the location data of citizen scientist submissions to NSF-NOIRLab, etc., were assembled as described in Supporting Information, Section 1.3–1.9.

Although not preregistered, this research was designed to investigate the authors' hypotheses about how environmental factors influence human psychology and behavior, and provides a foundation for future preregistered studies that should be conducted.

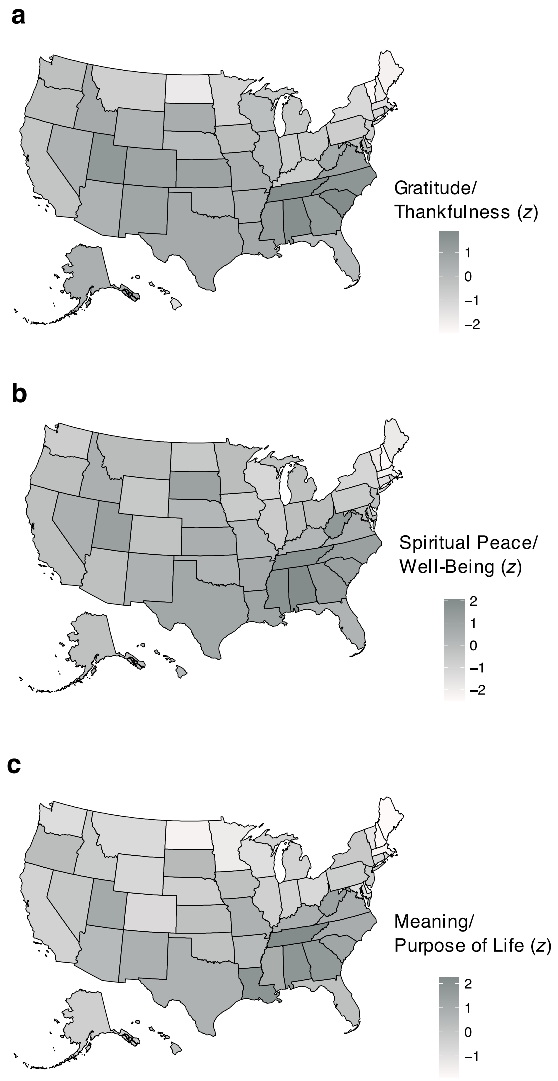

Supplementary Figure 1. State maps of the psychological emotions assessed in Pew 2014 survey in addition to wonder about the universe. Displayed are *z*-standardized scores for these three emotions, which were used as controls: (**a**) Gratitude/thankfulness, (**b**) Spiritual peace/well-being, (**c**) Meaning/purpose of life. See main text and Supplementary Information Section 1.2. for details.

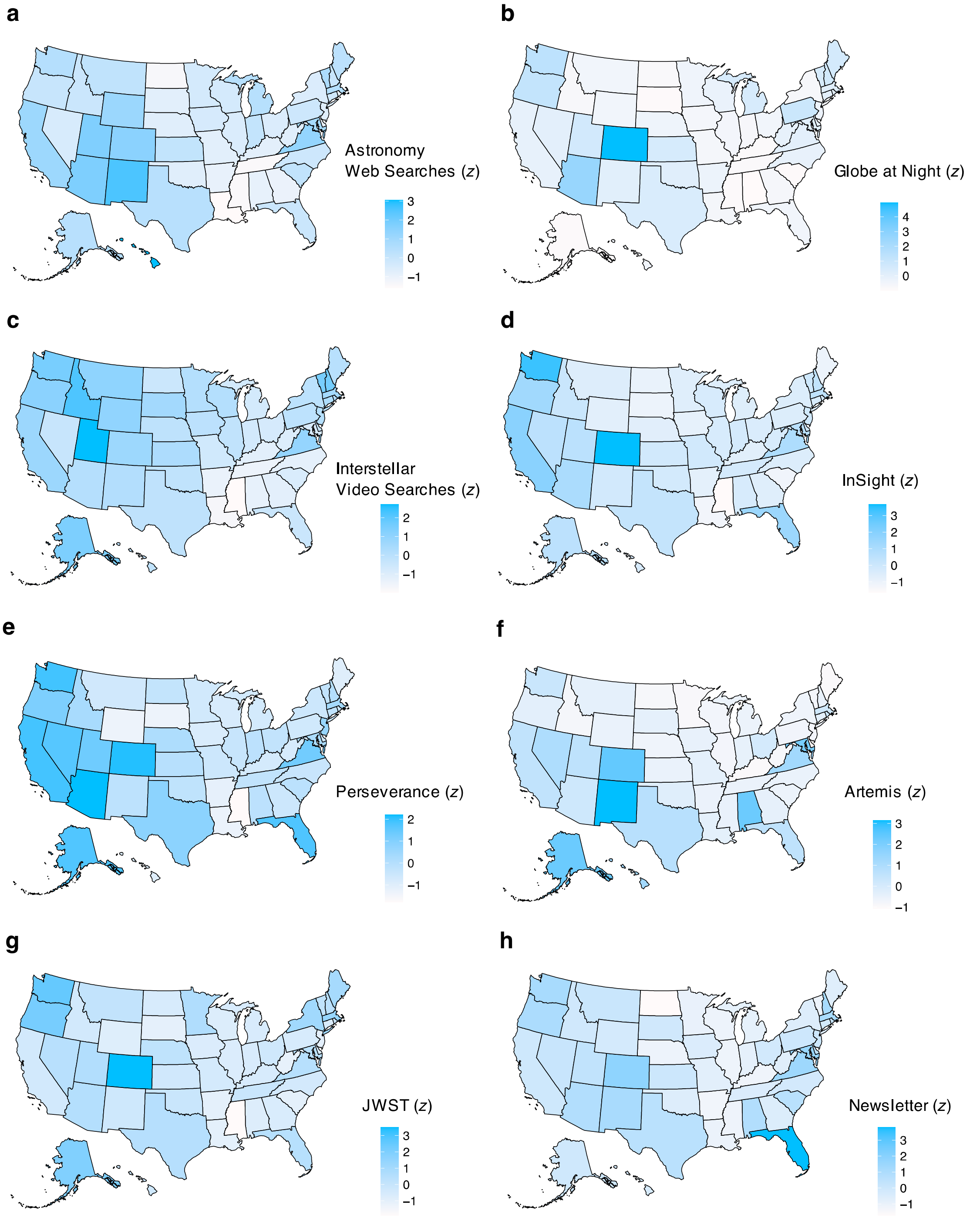

Supplementary Figure 2. State maps of each component measure of interest in astronomy. Displayed are *z*-standardized scores for eight components of the "behavioral interest in astronomy" composite measure, for each state. (**a**) Web searches for field of study "astronomy." (**b**) Submissions to Globe at Night. (**c**) Video searches for film *Interstellar*. (**d**) Names sent to Mars on *InSight*. (**e**) Names sent on *Perseverance*. (**f**) Artemis Generation astronaut applications. (**g**) Twitter (now X) followers of James Webb Space Telescope (JWST). (**h**) NASA Newsletter subscribers. See main text and Supplementary Information Sections 1.3-1.9. for details.

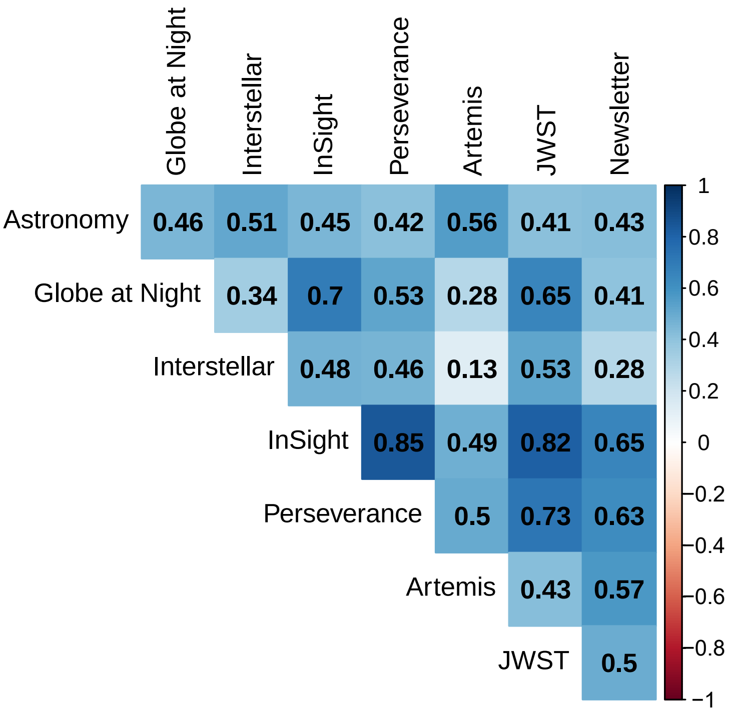

Supplementary Figure 3. Pearson zero-order correlations (r) among the 8 component measures. Displayed are the intercorrelations among the 8 components of the composite score of "behavioral interest in astronomy" (see main text for details). All measures are z-standardized. $r_{(df\,=\,48)\text{ critical value}} = 0.28$.

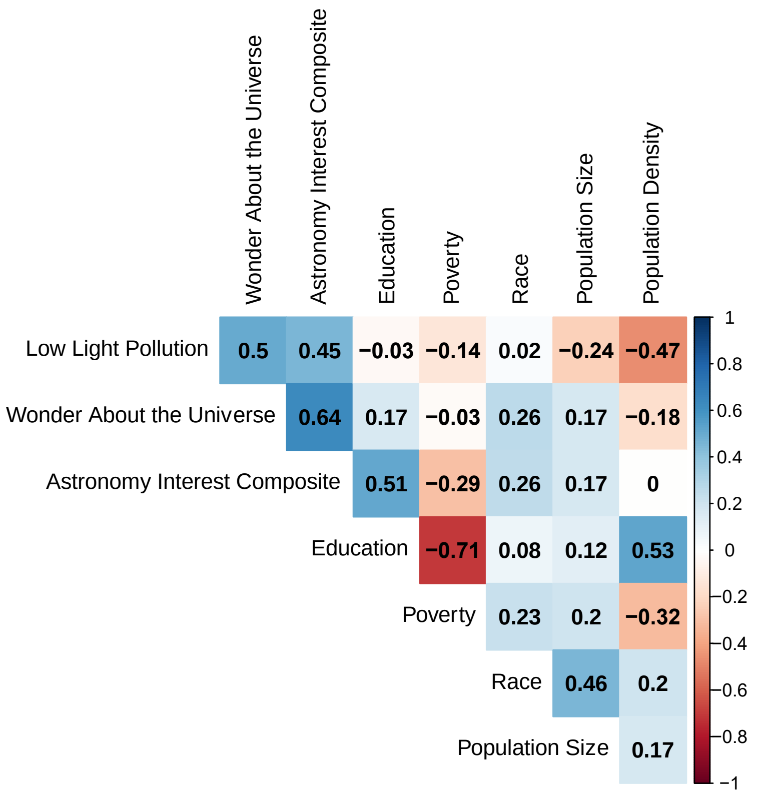

Supplementary Figure 4. Pearson zero-order correlations (r) among low light pollution, wonder about the universe, composite score of behavioral interest in astronomy, and demographic covariates (see Section 1.11. for demographic details). All measures are z-standardized. $r_{(df = 48)\ \text{critical value}} = 0.28$.

References

1. Falchi, F. *et al*. Light pollution in USA and Europe: The good, the bad and the ugly. *J. Environ. Manage.* **248**, 109227 (2019).
2. Falchi, F. *et al*. The new world atlas of artificial night sky brightness. *Sci. Adv.* **2**, e1600377 (2016).
3. Falchi, F. & Bará S. Light pollution is skyrocketing. *Science* **379**, 234–235 (2023).
4. Pew Research Center. *2014 Religious Landscape Study* https://www.pewforum.org/about-the-religious-landscape-study/ (2016).
5. Hayes, A. F. *Introduction to Mediation, Moderation, and Conditional Process Analysis: A Regression-Based Approach* (Guilford Press, 2018).
6. Prescod-Weinstein, C. *The Disordered Cosmos: A Journey into Dark Matter, Spacetime, & Dreams Deferred* (Bold Type, 2021).
7. Rees, M. *Our Cosmic Habitat* (Princeton University Press, 2008).
8. Ghose, S. *Her Space, Her Time: How Trailblazing Women Scientists Decoded the Hidden Universe* (Random House, 2023).
9. Loeb, A. *Extraterrestrial: The First Sign of Intelligent Life Beyond Earth* (Houghton Mifflin Harcourt, 2021).
10. Hawking, S. *Brief Answers to the Big Questions* (Bantam Books, 2018).
11. Johnson, K. E. Dark skies, bright kids: Helping young students reach for the stars. *University of Virginia Magazine* https://uvamagazine.org/articles/dark_skies_bright_kids (2012).
12. Master, A., Meltzoff, A. N. & Cheryan, S. Gender stereotypes about interests start early and cause gender disparities in computer science and engineering. *Proc. Natl. Acad. Sci. USA* **118**, e2100030118 (2021).
13. Master A., & Meltzoff, A. N. Building bridges between psychological science and education: Cultural stereotypes, STEM, and equity. *Prospects UNESCO*, **46**, 215–234 (2016).
14. Meltzoff, A. N., Kuhl, P. K., Movellan, J. & Sejnowski, T. J. Foundations for a new science of learning. *Science* **325**, 284–288 (2009).
15. Gopnik, A. & Meltzoff, A. N. *Words, thoughts, and theories* (MIT Press, 1997).
16. Gopnik, A., Meltzoff, A. N. & Kuhl, P. K. *The Scientist in the Crib: Minds, Brains, and How Children Learn* (William Morrow, 1999).
17. Master, A., Cheryan, S., Moscatelli, A. & Meltzoff, A. N. Programming experience promotes higher STEM motivation among first-grade girls. *J. Exp. Child Psychol.* **160**, 92–106 (2017).
18. Barragan, R. C., Brooks, R. & Meltzoff, A. N. Altruistic food sharing behavior by human infants after a hunger manipulation. *Sci. Rep.* **10**, 1785 (2020).
19. Barragan, R. C. & Meltzoff, A. N. Human infants can override possessive tendencies to share valued items with others. *Sci. Rep.* **11**, 9635 (2021).
20. Barragan, R. C. *et al*. Identifying with all humanity predicts cooperative health behaviors and helpful responding during COVID-19. *PLoS ONE* **16**, e0248234 (2021).
21. Cortes Barragan, R. C. & Meltzoff, A. N. Prosociality and health: Identification with all humanity is a replicable predictor of prosocial motivation for health behaviors. *Front. Psychol.* **13**, 1052713 (2023).
